# Cognitive Dysfunction after Heart Disease: A Manifestation of the Heart-Brain Axis

**DOI:** 10.1155/2021/4899688

**Published:** 2021-08-18

**Authors:** Chengyang Xu, Xueshu Tao, Xiaonan Ma, Rui Zhao, Zhipeng Cao

**Affiliations:** ^1^Department of Forensic Pathology, School of Forensic Medicine, China Medical University, No. 77, Puhe Road, Shenyang North New Area, Shenyang 110122, China; ^2^Department of Forensic Pathophysiology, School of Forensic Medicine, China Medical University, No. 77, Puhe Road, Shenyang North New Area, Shenyang 110122, China; ^3^Department of Pain Medicine, The First Hospital of China Medical University, Shenyang, China. No. 155 Nanjing North Street, Heping District, Shenyang, 110001, China

## Abstract

The functions of the brain and heart, which are the two main supporting organs of human life, are closely linked. Numerous studies have expounded the mechanisms of the brain-heart axis and its related clinical applications. However, the effect of heart disease on brain function, defined as the heart-brain axis, is less studied even though cognitive dysfunction after heart disease is one of its most frequently reported manifestations. Hypoperfusion caused by heart failure appears to be an important risk factor for cognitive decline. Blood perfusion, the immune response, and oxidative stress are the possible main mechanisms of cognitive dysfunction, indicating that the blood-brain barrier, glial cells, and amyloid-*β* may play active roles in these mechanisms. Clinicians should pay more attention to the cognitive function of patients with heart disease, especially those with heart failure. In addition, further research elucidating the associated mechanisms would help discover new therapeutic targets to intervene in the process of cognitive dysfunction after heart disease. This review discusses cognitive dysfunction in relation to heart disease and its potential mechanisms.

## 1. Introduction

The structure and the function of the heart and brain have been studied for several decades due to their important roles in life. With further understanding of the independent mechanisms of the two organs, scientists began to seek a connection between the heart and the brain, which was announced by Barrow in the middle of the 19th century ([1]).

The occurrence of brain disease, including cerebrovascular diseases, degenerative diseases of the nervous system, and infectious diseases of the central nervous system, can lead to myocardial injury and even cardiac dysfunction, which is described as the brain-heart axis [2–4]. On the other hand, the physiological activities and processes of the brain, such as cognitive ability and language capabilities, are directly affected by cardiac function, which is described as the heart-brain axis [5–8]. Cardiac dysfunction due to various causes, not only existing brain disease, can lead to new brain injury [8]. In this context, an increasing number of studies have focused on the connection between the heart and the brain.

Surprisingly, few studies have explored the effects of heart disease on brain function, one of the manifestations of the heart-brain axis [9]. Herein, cognitive dysfunction and its potential mechanisms are discussed.

## 2. Cognitive Dysfunction after Heart Disease

In 1982, the term “cardiogenic dementia” was quoted in an editorial of *The Lancet*, and it is characterized as patients suffering from cognitive decline after heart disease [10]. Up to 50% of patients with heart failure (HF) reportedly develop a certain degree of cognitive impairment, with 10% of patients suffering from more severe symptoms [11–13]. In contrast, only 26% of patients with treated chronic heart failure (CHF) develop vascular cognitive impairment [14]. The number of people grappling with heart disease has increased along with the aging population. Indeed, the incidence of HF increases with advancing age and is the most common cause of hospitalization in the United States, especially among people over 64 years old [15, 16]. Due to the lower cognitive reserve of the elderly, this population is more likely to develop cognitive dysfunction after heart disease [17].

As the main cause of HF, the high morbidity of coronary artery disease has led to the widespread implementation of coronary artery bypass grafting (CABG). In the United States alone, approximately 300,000 people undergo CABG each year [18]. In general, CABG surgery is safe in most cases; the complications mainly include postcardiac surgery syndrome, hypotensive brain injury, embolic stroke, visual defects, epilepsy, residual movement disorders, and rare peripheral nerve damage [19]. Most patients (80-90%) undergoing CABG surgery reportedly experience cognitive dysfunction at discharge [20]. Routine clinical examination found that the degree of cognitive impairment, measured by the Canadian Stroke Scale, Syndrom Kurztest, and psychiatric assessment, was often not obvious because it mainly manifested in the subcortex, involving executive dysfunction, depression, and anxiety. Over time, 42% of patients reportedly developed long-term (5 years) cognitive deficits [19]. Biomarkers of myocardial injury and cardiac dysfunction, such as cardiac troponin T (cTnT) and pro-N-terminal B-type natriuretic peptide (NT-proBNP), have been associated with lower cognitive ability among the elderly (over 60 years old), even in the absence of clinical disease [21–26]. This further illustrates that cardiac and cognitive functions are closely related.

The most obvious clinical manifestation of Alzheimer's disease (AD), a severe degenerative disease of the nervous system, is impaired cognitive function [27, 28]. Compared with a decreased total brain parenchymal volume of 0.32% per year in healthy aging individuals and brain parenchymal loss of 2% per year in AD patients, the annual loss of brain parenchymal volume was approximately 1% in patients with both high NT-proBNP levels and high carotid intima media thickness caused by reduced blood supply to the brain [29, 30]. In fact, biomarkers of myocardial injury and cardiac dysfunction have been repeatedly associated with changes in the brain structures of the elderly and the young, but only the elderly exhibit poor cognitive abilities [31]. This may be due to the higher cognitive reserve of young individuals, which can prevent the harmful effects of clinical heart disease on cognitive ability [17]. In view of these findings, it is hypothesized that the heart-brain axis runs through the processes of life [31].

## 3. Potential Mechanisms of Cognitive Dysfunction after Heart Disease

**Figure 1:**
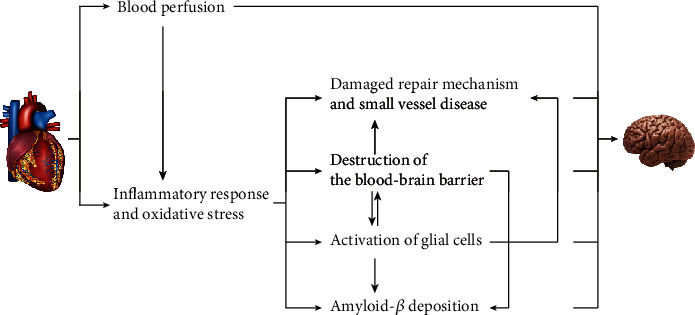
Potential mechanisms of cognitive dysfunction after heart diseases.

Many research groups have explored the potential mechanisms of cognitive impairment based on the important functions of the heart and brain and their interconnectivity. Among them, blood perfusion and inflammation are the two main mechanisms that have been explored (Figure 1).

### 3.1. Blood Perfusion

The brain is a highly energy-consuming organ, accounting for approximately 20% and 25% of the body's oxygen and glucose, respectively [32]. Its function depends on a sufficient supply of oxygen and energy via the bloodstream [33]. With fluctuations in blood flow and blood pressure throughout the body, multiple systems, including the cerebrovascular self-regulation mechanism, work together to maintain the cerebral blood flow within a stable range [34]. The heart is where cerebral perfusion pressure is generated, and intra- and extracranial blood vessels change cerebral perfusion, as they deliver blood to the brain [35]. The brain receives approximately 15% of the cardiac output [36].

Hypoperfusion due to low cardiac output is a key factor in cardiovascular disease, which accelerates changes in the brain structure [37, 38]. In the case of long-term systemic hypoperfusion, the blood flow regulation mechanism may not be able to compensate to protect the brain, resulting in insufficient cerebral perfusion, which is associated with impaired brain function [32]. Decreased cerebral blood flow in patients with HF has been associated with increased prevalence of cognitive dysfunction, and even a subclinical decrease in cardiac output has been associated with impaired cognitive function [39–41]. Patients with CHF are more prone to brain parenchymal loss, especially gray matter, due to low cerebral blood flow [42–44]. This is because the higher metabolic demand of gray matter requires more than two-thirds of the cerebral blood flow [32, 36]. Reduced gray matter volume was reportedly associated with impairment of various cortical areas involved in executive cognitive function [45]. Furthermore, surgery to improve cardiac function, such as heart transplantation or resynchronization, has been shown to help develop cognitive function [46, 47]. Therefore, cardiac dysfunction can lead to hypoperfusion and ultimately cause cognitive impairment.

Vascular damage in the brain itself, such as small vessel disease, may also increase the vulnerability of the brain to changes in perfusion pressure [27]. In contrast, changes in cerebral blood flow homeostasis can promote clinical or subclinical brain injury by aggravating microvascular damage. For example, fluctuations in cerebral perfusion can lead to changes in microvascular structure, expression of vascular cell receptors, microvascular permeability, and vascular remodeling [48, 49].

Increasing evidence supports the role of blood perfusion in the pathogenesis of AD [27, 28]. Early AD without symptoms manifests as cognitive decline caused by hypoperfusion, and increasing cerebral blood flow has been shown to improve AD symptoms [28]. This also supports the close connection between blood perfusion and cognitive decline.

In the case of cerebral hypoperfusion, the metabolic activity of the whole brain is significantly reduced [50]. Specifically, the metabolic level of the parietal area in patients with HF was reportedly reduced compared with that in healthy volunteers [51, 52]. However, in HF patients with hibernating myocardium and impaired global left ventricular function, the metabolism of the frontal lobe area was compensatorily increased, while glucose metabolism of the hippocampus and parahippocampus, which are vulnerable to dementia, was decreased, leading to memory loss [50, 53]. These differential changes in metabolic activity were related to self-regulation efficiency in different areas [54]. As part of the neocortex, the frontal lobe may have higher self-regulation efficiency and sensitivity than the original cortex, including the hippocampus and parahippocampus [55]. Therefore, when cerebral blood flow begins to decrease, the forehead area may be compensated first to maintain a certain degree of energy metabolism.

Except for heart diseases, such as myocarditis and myocardial infarction (MI) that directly lead to inflammation, hypoperfusion can cause inflammation of brain tissues in diseases with decreased cardiac function, such as HF [56]. In response to hypoperfusion injury or interruption of perfusion, cell respiration is quickly exhausted, and adenosine triphosphate (ATP) is consumed and regenerated without compensation. Subsequently, reactive oxygen species (ROS) are produced [57, 58]. Specific inflammatory changes are discussed in the next section.

Aerobic exercise has demonstrated amelioratory effects on the blood supply to the brain. On the basis of the mechanism discussed above, aerobic exercise is beneficial for cardiac disease patients with cognitive impairment. Regular aerobic physical activity can increase the blood flow in the prefrontal cortex, promote the formation of blood vessels in the frontal cortex, and increase the concentration of the vascular endothelial growth factor [59]. Moreover, long-term continuous aerobic exercise can promote cardiovascular health by increasing vagal tone and reducing sinus node sympathetic nerve activity [60]. Tanne et al. reported that HF patients with the New York Heart Association (NYHA) functional class III status and an ejection fraction of 35% demonstrated improved selective attention and mental exercise speed after long-term aerobic exercise [15].

### 3.2. Inflammatory Response and Oxidative Stress

The inflammatory response and oxidative stress are usually interdependent and may be potentially associated with brain dysfunction after heart disease [61, 62]. Studies have reported that inflammation occurs in the brain after MI. However, local inflammation of the skeletal muscle has no effect on the heart and brain, indicating that the connection between the heart and the brain is closer than that of other organs when the heart is injured [63]. On the one hand, several heart diseases, such as myocarditis and MI, will directly cause inflammation; on the other hand, hypoperfusion caused by diseases with decreased heart function will also lead to brain inflammation [56]. In addition, decreased cardiac function can change inflammatory marker levels in the brain [64]. The expression of inflammatory genes, such as toll-like receptor- (TLR-) 4, tumor necrosis factor- (TNF-) *α*, and interleukin- (IL-) 6, was significantly upregulated in the cortex and hippocampus in a mouse model of HF [65]. As for the pathological mechanism of HF, the landmark event in its early stage is the appearance of a large number of proinflammatory factors in the heart and peripheral circulation, such as TNF-*α*, IL-1, IL-6, IL-8, and monocyte chemoattractant protein- (MCP-) 1, mainly derived from neutrophils, monocytes, and macrophages [66]. These proinflammatory factors reach the brain via the bloodstream, causing inflammation in the brain.

TNF-*α*, which is considered the main regulator of the brain's proinflammatory response, can cause cell damage and death by increasing neurotoxicity via neuronal glutamate [67, 68]. Recent studies have shown that the ASK1-p38-TNF-*α* pathway is involved in the destruction of tight junctions soon after chronic cerebral hypoperfusion [69]. Insufficient cerebral perfusion affects the level of IL-1*β* in the central nervous system, which is an important regulator of the inflammatory cascade [70]. TNF-*α* and IL-1*β* in the ischemic brain can reportedly induce the production of IL-6, and increased IL-6 levels in the serum and cerebrospinal fluid are related to cognitive impairment [71]. Furthermore, the presence of IL-1*β* and TNF-*α* can upregulate the expression and function of CD73, which plays a protective role in the occurrence of white matter lesions induced by cerebral hypoperfusion and cognitive impairment by regulating the activation of glial cells and expression of proinflammatory cytokines [72]. Specifically, upregulation of CD73 expression and activity in the corpus callosum after chronic hypoperfusion mediates the production of adenosine, which can inhibit the release of inflammatory cytokines IL-1*β*, IL-6, and TNF-*α*. Therefore, CD73 plays a key role in the counter-regulatory feedback mechanism, helping to relieve inflammation [69].

The inflammatory response and oxidative stress mainly cause cognitive dysfunction through damage to the self-repair mechanism, destruction of the blood-brain barrier (BBB), and activation of glial cells [61, 73, 74]. Among them, the damaged BBB and activated glial cells will also affect each other, leading to deposition of amyloid-*β* (A*β*) and causing damage to the cognitive function of the brain [75–77].

In view of the importance of the inflammatory response and oxidative stress, anti-inflammatory or antioxidant therapies have become an innovative clinical approach [78]. Naturally, the optimal drug should simultaneously treat heart disease and cognitive dysfunction, and angiotensin-converting enzyme inhibitors (ACEIs) have received the most attention. Among them, enalapril inhibits the proinflammatory activity of angiotensin II [79, 80]. Continuous treatment with enalapril has been shown to reduce acute and chronic inflammation in the heart and brain [63]. Animal experiments suggested that the effectiveness of ACEIs on neuroinflammation after MI was based on acute application, since late application missed the acute anti-inflammation stage and was not beneficial for alleviating chronic neuroinflammation [81]. As for other noncardiac treatment drugs, translocator protein inhibitors, such as 4′-chlordiazepam, are believed to modulate neuroinflammation and exert cardioprotective effects during ischemia-reperfusion injury through antioxidant mechanisms [82]. Nonsteroidal anti-inflammatory drugs have also demonstrated a certain effectiveness against amyloid deposition [83].

#### 3.2.1. Damaged Repair Mechanism and Development of Small Vessel Disease

The inflammatory response and oxidative stress are believed to play important roles in neurovascular dysfunction. Oxidative stress can cause endothelial dysfunction, BBB disruption, and cytokine production [61]. In turn, inflammation can enhance oxidative stress by upregulating the expression of enzymes that produce ROS and downregulating antioxidant defense capabilities [62]. Further, the inflammatory response and oxidative stress disrupt the repair mechanism of damaged white matter by interfering with the proliferation, migration, and differentiation of oligodendrocyte progenitor cells [73, 84]. The loss of growth factors, which are produced by the brain, reportedly occurs in vascular cognitive impairment, further damaging the repair mechanism and aggravating cognitive impairment [74].

Apolipoprotein E (ApoE) subsequently affects neurovascular units through pericytes, causing changes in microvascular flow and triggering neurodegenerative changes [85]. Cerebral arterioles are particularly vulnerable to these changes, promoting the development of small vessel diseases, such as cerebral amyloid angiopathy and arteriole sclerosis [9]. Small vessel diseases lead to weakened cerebral vasoconstriction, which in turn promotes cerebral blood pressure changes [86, 87]. Combined, these factors eventually lead to cognitive impairment, stroke symptoms, and changes in brain structure, such as white matter lesions, lacunar infarction, and substantial bleeding [9].

Overall, the inflammatory response and hypoperfusion can damage the brain repair mechanism, which in turn promote the occurrence of small vessel diseases.

#### 3.2.2. Destruction of the Blood-Brain Barrier

Abnormal function of the BBB, including activated endothelial cells, destroyed pericytes, and abnormal endothelial-endothelial and endothelial-pericyte connections, occupies an important position in the pathogenesis of cognitive dysfunction after heart disease [35]. Inflammation and ROS are thought to be involved in these changes, since the generation of ROS destroys the ion gradient [88]. Endothelial cells, the main component of the BBB, have been shown to be damaged by an increase in intracellular calcium ion levels [89]. In addition, pericytes are reportedly involved in regulating BBB function and are highly likely to cause destructive inflammation [85, 90]. The breakdown of the BBB is caused by activation of the proinflammatory pathway of pericytes, involving cyclophilin A-nuclear factor-*κ*B-matrix-metalloproteinase-9 [69, 74]. Cerebral endothelial tight junction proteins, such as claudin-5, play a key role in maintaining the BBB [91]. Thus, decreased levels of claudin-5 in the corpus callosum destroy the BBB via the ASK1-p38-TNF-*α* pathway. Animal experiments have demonstrated that the use of compound K811, a specific ASK1 inhibitor, can prevent cognitive impairment in a mouse model of hypoperfusion [72]. ASK1 may be a new target molecule for the treatment of cognitive dysfunction caused by cerebral hypoperfusion.

Damage to the BBB increases its permeability, causing leakage of plasma components and exchange of substances [92]. After the occurrence of hypoperfusion, immunoglobulin (Ig) G leakage from the corpus callosum indicated BBB rupture [93]. This leakage occurred when glial cells, including astrocytes and microglia, were not observed to be activated [94]. Moreover, the level of proinflammatory markers was related to the degree of BBB rupture [95]. Notably, the complete BBB plays an important role in preventing extravasation of toxic factors from circulating to the brain parenchyma. For instance, peripheral blood-derived substances, such as thrombin, can aggravate central nervous system inflammation [96, 97]. BBB dysfunction thus further exacerbates microvascular damage, promotes secondary inflammation, and damages blood vessel tension, leading to luminal stenosis and tissue ischemia [98].

Generally, destruction of the BBB by ROS and inflammation after heart disease will lead to further aggravation of inflammation and promote contact of neurotoxic proteins with neurons. The relationship between BBB destruction and immune cell activation is described later.

#### 3.2.3. Activation of Glial Cells

After inflammation occurs, astrocytes are activated slightly earlier than microglia [63]. The activation of astrocytes after MI is directly related to the inflammation induced by infarction, and may be caused by proinflammatory cytokines [75]. ^11^C-methionine uptake can help reflect inflammation in the brain [99]. Neutrophils and proinflammatory monocytes display higher rates of methionine uptake, which affects the activation of astrocytes [100]. In addition, active astrocytes may be neurotoxic and produce specific inflammatory cytokines and ROS [75].

The increased permeability of the BBB also enhances the adhesion and migration of monocytes from the periphery to the central nervous system, and then differentiation into microglia to promote neuroinflammation [76]. As the main agonist of neuroinflammation, the production of microglia is the central nervous system's immune response to local injury or systemic activation [101]. Neuroinflammation is considered key to the progression of AD [102], in which microglia are activated by amyloid deposition and exert neurotoxic effects, thereby further aggravating inflammation and expanding amyloid deposition [77]. Subsequently, the activated microglia produce a large amount of proinflammatory cytokines, leading to cumulative damage to the tissue [103]. Miyanohara et al. reported that proinflammatory cytokines (such as IL6 and TNF-*α*) and obvious tissue damage were significantly detected in the white matter of a mouse model of chronic cerebral hypoperfusion with BCAS [104]. Proinflammatory cytokines also play an important role in oligodendrocyte toxicity [105]. This relationship is maturation-dependent, i.e., proinflammatory cytokines are more toxic to immature oligodendrocytes than mature oligodendrocytes [106].

To study the mechanism of inflammation in depth, Miyanohara et al. confirmed that transient receptor potential melastatin 2 (TRPM2), a Ca2+ permeable channel, participated in cognitive impairment related to chronic cerebral hypoperfusion by enhancing the production of cytokines, leading to subsequent white matter damage [104, 107]. Specifically, TRPM2, expressed in microglia/macrophages, mediated the upregulation of proinflammatory cytokines in white matter [104]. In the regulation of TRPM2, ROS was considered an endogenous agonist of TRPM2, and high concentrations of hydrogen peroxide induced TRPM2-mediated neuronal death [108–110].

Apart from TRPM2, IL1R, CX3CRI, leptin, and tamoxifen may also be involved in regulation of the inflammatory response. Zhou et al. reported that the loss of IL1R expression improved white matter damage and promoted the migration of oligodendrocyte precursor cells from the subventricular zone [111]. Further, CX3CR1 silencing in microglia reduced cytokine release, white matter damage, and cognitive impairment [112]. Leptin reportedly increased the expression of proinflammatory cytokines induced by hypoxia in BV2 microglia [113]. Du et al. reported that leptin receptor deletion protected mice from cognitive dysfunction induced by cerebral hypoperfusion and white matter damage by inhibiting glial cell activation and the proinflammatory response, which promoted the expression of anti-inflammatory cytokines in white matter and activation of M2 microglia [114]. Tamoxifen significantly reduced the activation and inflammatory response of microglia, facilitated the polarization of microglia to the M2 phenotype, enhanced the proliferation and differentiation of oligodendrocyte precursor cells, and reduced the expression of TNF-*α* and IL-1*β* [115].

In short, inflammation and destruction of BBB permeability after heart disease promote the activation of glial cells, leading to neuroinflammation, which is considered an important factor in the progression of AD.

#### 3.2.4. Amyloid-*β* Deposition

Although HF and cognitive dysfunction in patients with AD have long been considered independent factors, an increasing number of studies have confirmed that they may be related, with similarities such as common risk factors and similar epidemiological stratification [88]. Moreover, AD-related neuropathology was reported in a rat model after acute blood perfusion ceased, including A*β* deposition in the hippocampus, endothelium, and neocerebral cortex, which led to neuronal death [116].

Under normal circumstances, A*β* is removed from the brain through the BBB, the interstitial bulk flow clearance (glymphatic) system, or meningeal lymphatic vessels [117, 118]. Under chronic hypoperfusion, these drainage systems are damaged, and A*β* accumulates in the perivascular space [119]. In the process of A*β* deposition, new evidence suggests that immune mediators also contribute to disease progression, especially in microglia [76]. Such scavenger cells play a role in both the inflammatory cascade and A*β* clearance in the brain [120]. Chronic low blood flow and glucose transport conditions result in only a few activated microglia in the area around the A*β* plaque, accompanied by impaired function [76]. Thus, the ability of microglia to eliminate A*β* is further reduced, providing an ideal environment for accumulation, aggregation, and further formation of plaques. In addition, active astrocytes also express high levels of amyloid precursor protein [121]. Therefore, astrocyte activation induced by heart disease may lead to a vicious cycle of amyloid production and further neuroinflammation [122].

Studies with mouse models of AD and AD patients reported that A*β*, in turn, caused oxidative imbalance through A*β* accumulation in the mitochondria, which aggravated mitochondrial dysfunction, ROS production, and overall oxidative imbalance [123, 124]. The cycle was further exacerbated by the inflammatory response and oxidative stress causing vasoconstriction and promoting destruction of the BBB. A*β* accumulation in the brain also reportedly has a profound effect on blood vessels, thereby promoting neurodegenerative processes [88].

In summary, under the condition of chronic hypoperfusion, damage to the drainage system and decreased ability of glial cells to clear A*β* lead to the accumulation of A*β*, eventually causing cognitive impairment. Interestingly, HF with preserved ejection fraction can promote cognitive dysfunction in AD and vice versa [88]. Overall, HF and cognitive dysfunction are interrelated, and their common pathogenesis may be systemic [125]. In view of the important roles of the heart and brain, the relationship between them must be taken seriously.

## 4. Future Direction

Cognitive dysfunction occurs in a large number of patients with heart disease, causing a great burden to families and the healthcare system. However, systematic research still needs to be conducted. Recently, increasing evidence has shown that extracellular vesicles are involved in molecular transport within and between organs, including interactions between the heart and brain [126]. In the brain, exosomes have important physiological functions, such as promoting signal transmission between neurons and glial cells and participating in A*β* metabolism [127, 128]. At present, exosomes, as members of extracellular vesicles, are also known to participate in the heart-brain axis. Sun et al. described a new mechanism of brain damage originating from the heart, in which high levels of microRNA- (miR-) 1 were produced in the ischemic and marginal regions of the heart in a mouse model of MI. Transported by exosomes from the heart, miR-1 reached the hippocampus, reduced the expression of tubulin polymerization-promoting protein, and affected the stability of microtubules in neurons [129]. Furthermore, the expression of miR-133b, which is responsible for the development of midbrain dopaminergic neurons, was reportedly decreased in circulation after MI [130].

In addition to probing the mechanisms of cognitive dysfunction after heart disease, the clinical application of these mechanisms is also an important point for scientists and doctors. The impact on cognitive function of drugs used to treat heart disease needs to be carefully evaluated, especially new therapies such as sacubitril/valsartan. Theoretically, neprilysin inhibition reduces A*β* degradation in the central nervous system, thereby increasing the risk of AD [131]. ACEI drugs pose similar risks, as they are involved in the process of converting amyloid-*β*1-42 into A*β*1-40. ACEIs block this process and increase the deposition of A*β*1-42 in the brain [59]. However, the specific role of these drugs in the process of cognitive dysfunction remains to be further elucidated.
